# Supplementary material for: Eugenol, Isoeugenol, Thymol, Carvacrol, and Ester Derivatives as an Ecofriendly Option to Control Glomerella Leaf Spot and Bitter Rot on Apple
Source: Plants (Basel). 2024 Nov 14;13(22):3196. doi: 10.3390/plants13223196 (PMC11598785; doi:10.3390/plants13223196)

SUPPLEMENTARY MATERIALS

Supplementary tables

Table S1 Estimates of relative risk for the expression of symptoms of apple bitter rot caused by *C. nymphaeae*, estimated by the Cox semiparametric model, followed by 95% confidence intervals for fruit.

Experiment 1

Treatment*	Incubation period (days)**	Relative risc	CI (95%)***		Latent period (days)	Relative risc	CI (95%)***	
			LL	US			LL	US
Control	3				10			
**CvAc**	4	0.764	0.576	1.014	**11**	**0.579**	**0.364**	**0.922**
**EgAc**	**4**	**0.476**	**0.348**	**0.651**	**10**	**0.332**	**0.198**	**0.558**
TmAc	4	0.853	0.645	1.127	10	0.794	0.511	1.232
**CvBt**	4	0.884	0.671	1.166	**10**	**0.569**	**0.356**	**0.911**
Cv	4	0.900	0.685	1.183	10	0.786	0.510	1.212
DMSO	3	1.008	0.774	1.312	10	0.788	0.514	1.207
**Eug**	**4**	**0.738**	**0.554**	**0.983**	**11**	**0.478**	**0.292**	**0.784**
Ieg	3	0.966	0.736	1.267	10	0.727	0.469	1.129
**Mzeb 100**	3	0.970	0.742	1.268	**11**	**0.494**	**0.305**	**0.799**
**Mzeb 200**	**4**	**0.750**	**0.564**	**0.999**	**10**	**0.594**	**0.375**	**0.941**
TmBt	4	0.806	0.606	1.073	10	0.655	0.413	1.038
Tm	4	0.824	0.625	1.086	10	0.699	0.450	1.085

Experiment 2

Treatment*	Incubation period (days)**	Relative risc	CI(95%)***		Latent period (days)	Relative risc	CI (95%)***	
			LL	US			LL	US
Control	7				16			
CvAc	7	1.179	0.882	1.577	14	0.929	0.573	1.506
EgAc	7	1.265	0.950	1.684	16	0.680	0.403	1.148
TmAc	6	1.301	0.978	1.732	14	1.089	0.682	1.741
CvBt	6	1.378	1.040	1.828	14	1.355	0.866	2.121
Cv	5	1.508	1.142	1.992	16	0.936	0.577	1.516
DMSO	5	1.115	0.832	1.495	14	0.960	0.594	1.550
**Eug**	10	0.820	0.597	1.128	**>16**	**0.287**	**0.145**	**0.567**
Ieg	7	1.112	0.829	1.493	>16	0.601	0.351	1.027
Mzeb 100	8	1.316	0.989	1.750	16	0.909	0.558	1.479
Mzeb 200	5	1.426	1.077	1.890	14	0.714	0.426	1.196
TmBt	7	0.945	0.698	1.281	14	1.188	0.754	1.873
**Tm**	7	1.113	0.830	1.495	**>16**	**0.400**	**0.218**	**0.736**

**Ex vivo* assay carried out with apple tree fruits, inoculated with *C. nymphaeae*, previously treated with Eugenol (Eg); Eugenyl acetate (EgAc); Isoeugenol (Ieg); Thymol (Tm); Thymol acetate (TmAc); Thymol butyrate (TmBt); Carvacrol (Cv); Carvacryl acetate (CvAc); Carvacryl butyrate (CvBt); Dimethyl solfoxide (DMSO) and Manzate 800© two concentrations (Mzeb 100 and Mzeb 200).
**Incubation period is the number of days between the inoculation (contact of the pathogen with the apple leaf) and the symptoms expression on at least 50% of the sample (inoculated leaf).
***CI: confidence interval (95%); LL: lower limits; US: upper limit.

Table S2. Estimates of relative risk for the expression of symptoms of apple bitter rot caused by *C. chrysophillum* estimated by the Cox semiparametric model, followed by 95% confidence intervals for fruit.

Experiment 1

Treatment*	Incubation period (days)**	Relative risc	CI(95%)***		Latent period (days)	Relative risc	CI(95%)***	
			LL	US			LL	US
Control	3				11			
**CvAc**	**5**	**0.497**	**0.358**	**0.691**	**12**	**0.228**	**0.115**	**0.453**
**EgAc**	**4**	**0.732**	**0.548**	**0.979**	**10**	**0.500**	**0.294**	**0.850**
TmAc	4	0.934	0.707	1.233	10	0.683	0.414	1.124
CvBt	3	0.860	0.651	1.138	**11**	**0.570**	**0.352**	**0.990**
**Cv**	**4**	**0.732**	**0.548**	**0.979**	10	0.728	0.446	1.189
DMSO	3	0.819	0.615	1.090	10	0.664	0.401	1.098
Eug	3	0.990	0.749	1.308	10	1.094	0.686	1.747
**Ieg**	**4**	**0.734**	**0.548**	**0.982**	**12**	**0.405**	**0.227**	**0.721**
Mzeb 100	3	0.909	0.688	1.201	**11**	**0.549**	**0.327**	**0.922**
Mzeb 200	3	0.890	0.673	1.178	10	1.026	0.646	1.629
**TmBt**	**4**	**0.709**	**0.522**	**0.965**	**14**	**0.547**	**0.315**	**0.950**
Tm	3	0.884	0.666	1.174	11	0.800	0.486	1.317

Experiment 2

Treatment*	Incubation period (days)**	Relative risc	CI(95%)***		Latent period (days)	Relative risc	CI(95%)***	
			LL	US			LL	US
Control	7				16			
CvAc	8	1.036	0.769	1.396	16	0.892	0.561	1.417
EgAc	7	1.373	1.040	1.814	14	1.159	0.751	1.789
TmAc	6	1.411	1.070	1.861	11	1.909	1.282	2.843
CvBt	8	1.135	0.849	1.517	11	1.609	1.066	2.430
Cv	7	1.124	0.842	1.501	14	0.915	0.578	1.449
DMSO	8	0.877	0.647	1.188	16	0.786	0.491	1.258
**Eug**	9	0.979	0.726	1.320	**16**	**0.379**	**0.211**	**0.679**
**Ieg**	**11**	**0.725**	**0.528**	**0.997**	14	0.752	0.468	1.209
Mzeb 100	9	0.984	0.730	1.325	11	1.305	0.854	1.994
Mzeb 200	6	1.318	0.997	1.743	16	0.791	0.492	1.271
TmBt	8	1.122	0.840	1.499	14	1.267	0.826	1.944
Tm	6	1.428	1.082	1.885	14	1.663	1.101	2.511

**Ex vivo* assay carried out with apple tree fruits, inoculated with *C. chrysophillum*, previously treated with Eugenol (Eug); Eugenyl acetate (EgAc); Isoeugenol (Ieg); Thymol (Tm); Thymol acetate (TmAc); Thymol butyrate (TmBt); Carvacrol (Cv); Carvacryl acetate (CvAc); Carvacryl butyrate (CvBt); Dimethyl solfoxide (DMSO) and Manzate 800© two concentrations (Mzeb 100 and Mzeb 200).
**Incubation period is the number of days between the inoculation (contact of the pathogen with the apple leaf) and the symptoms expression on at least 50% of the sample (inoculated leaf).
***CI: confidence interval (95%); LL: lower limits; US: upper limit.

Table S3. Estimates of relative risk for the expression of symptoms of Glomerella Leaf Spot with inoculation of mixture of *Colletotrichum nymphaeae* and *Colletotrichum chrysophillum*, estimated by the Cox semiparametric model, followed by 95% confidence intervals for leaf.

Experiment 1								
**Treatment***	**Incubation period (days)****	**Relative risc**	**CI(95%)*****		**Latent period (days)**	**Relative risc**	**CI(95%)*****	
			**LL**	**US**			**LL**	**US**
Control	6				12			
CvAc	>13	0,084	0,033	0,212	>13	0,112	0,033	0,378
EgAc	>13	0,362	0,214	0,613	>13	0,231	0,092	0,577
TmAc	>13	0,369	0,218	0,625	>13	0,277	0,117	0,656
CvBt	>13	0,000	0,000	inf	>13	0,000	0,000	inf
Cv	>13	0,050	0,016	0,162	>13	0,037	0,005	0,276
Eug	>13	0,050	0,016	0,162	>13	0,037	0,005	0,276
Ieg	>13	0,383	0,229	0,643	>13	0,153	0,052	0,449
Mzeb 100	>13	0,137	0,065	0,292	>13	0,192	0,072	0,514
Mzeb 200	>13	0,033	0,008	0,137	>13	0,036	0,005	0,272
TmBt	>13	0,119	0,054	0,266	>13	0,076	0,018	0,325
Tm	>13	0,331	0,192	0,572	>13	0,231	0,093	0,578

Experiment 2								
**Treatment***	**Incubation period (days)****	**Relative risc**	**CI(95%)*****		**Latent period (days)**	**Relative risc**	**CI(95%)*****	
			**LL**	**US**			**LL**	**US**
Control	4				12			
CvAc	>13	0,072	0,029	0,180	>13	0,049	0,012	0,211
EgAc	>13	0,000	0,000	inf	>13	0,000	0,000	inf
TmAc	>13	0,165	0,086	0,317	>13	0,076	0,023	0,256
CvBt	>13	0,103	0,047	0,226	>13	0,025	0,003	0,183
Cv	>13	0,057	0,021	0,159	>13	0,000	0,000	inf
Eug	4	0,599	0,391	0,918	>13	0,140	0,053	0,367
Ieg	>13	0,231	0,130	0,410	>13	0,000	0,000	inf
Mzeb 100	>13	0,000	0,000	inf	>13	0,000	0,000	inf
Mzeb 200	>13	0,103	0,047	0,226	>13	0,050	0,012	0,211
TmBt	>13	0,163	0,085	0,313	>13	0,025	0,003	0,183
Tm	>13	0,000	0,000	inf	>13	0,000	0,000	inf

*Ex vivo test carried out with detached leaves of apple seedlings, inoculated with a mixture of equivalent parts of *Coletotrichun nymphaeae* and *Colletotrichum chrysophillum*, previously treated with Eugenol (Eug); Eugenyl acetate (EgAc); Isoeugenol (Ieg); Thymol (Tm); Thymol acetate (TmAc); Thymol butyrate (TmBt); Carvacrol (Cv); Carvacryl acetate (CvAc); Carvacryl butyrate (CvBt) and Manzate 800© two concentrations (Mzeb 100 and Mzeb 200).

**Incubation period is the number of days between the inoculation (contact of the pathogen with the apple leaf) and the symptoms expression on at least 51% of the sample (inoculated leaf).

***CI: confidence interval (95%); LL: lower limits; US: upper limit.

Supplementary spectral data

The yields and spectral data obtained for each compound are available above.

4-allyl-2-methoxyphenyl acetate (Eugenyl acetate, EgAc). Yield, 90%: ^{1}H NMR (200 MHz, $CDCl_3$): δ (ppm) 6.95 (d, J = 8.0 Hz, 1H), 6.81 – 6.72 (m, 2H), 5.96 (ddt, J = 17.0; 10.2; 6.7 Hz, 1H), 5.18 – 5.4 (m, 2H), 3.82 (s, 3H), 3.38 (d, J = 6.7 Hz, 2H), 2.31 (s, 3H). ^{13}C NMR (50 MHz, $CDCl_3$): δ 169.3; 150.7; 139.0; 137.8; 137.0; 122.4; 120.6; 116.1; 112.6; 55.7; 40.0; 20.7. MS (EI) m/z (rel. int.): 206 (4.24%), 164 (84.18%), 149 (35.12%), 104 (34.51%), 103 (39.67%), 91 (65.79%), 77 (62.49%), 55 (35.02%), 43 (100.00%).

(*E*)-2-methoxy-4-(prop-1-en-1-yl) phenyl acetate (Isoeugenyl acetate, IegAc). Yield, 85%: ^{1}H NMR (200 MHz, $CDCl_3$): δ (ppm) 6.98 – 6.86 (m, 3H), 6.43 – 6.31 (m, 1H), 6.18 (dq, J = 15.7; 6.2 Hz, 1H), 3.84 (s, 3H), 2.31 (s, 3H), 1.88 (dd, J = 6.3; 1.2 Hz, 3H). ^{13}C NMR (50 MHz, $CDCl_3$): δ 169.2; 150.9; 138.4; 137.0; 130.4; 126.1; 122.6; 118.3; 109.5; 55.7; 20.7; 18.4. MS (EI) m/z (rel. int.): 206 (0.32%), 103 (13.08%), 91 (37.22%), 77 (31.64%), 65 (24.63%), 63 (14.32%), 55 (24.08), 51 (18.40%), 43 (100.00%).

2-isopropyl-5-methylphenyl acetate (Thymol acetate, TmAc). Yield, 85%: ^{1}H NMR (200 MHz, $CDCl_3$): δ (ppm) 7.20 (d, J = 7.9 Hz, 1H), 7.02 (dd, J = 7.9; 1.1 Hz, 1H), 6.81 (d, J = 1.1 Hz, 1H), 2.97 (hept, J = 6.9 Hz, 1H), 2.31 (s, 6H), 1.19 (d, J = 6.9 Hz, 6H). ^{13}C NMR (50 MHz, $CDCl_3$): δ 169.7; 147.9; 137.0; 136.5; 127.1; 126.4; 122.7; 27.1; 23.0; 20.9; 20.8. MS (EI) m/z (rel. int.): 192 (11.18%), 150 (50.55%), 136 (15.16%), 135 (100.00%), 115 (8.96%), 107 (8.47%), 91 (13.01%).

5-isopropyl-2-methylphenyl acetate (Carvacryl acetate, CvAc). Yield, 98%: ^{1}H NMR (200 MHz, $CDCl_3$): δ (ppm) 7.14 (d, J = 7.8 Hz, 1H), 7.01 (dd, J = 7.8; 1.6 Hz, 1H), 6.86 (d, J = 1.3 Hz, 1H), 2.87 (hept, J = 6.9 Hz, 1H), 2.31 (s, 3H), 2.13 (s, 3H), 1.23 (d, J = 6.9 Hz, 6H). ^{13}C NMR (50 MHz, $CDCl_3$): δ 169.3; 149.2; 148.0; 130.8; 127.1; 124.18; 119.7; 33.5; 23.8; 20.8; 15.7. MS (EI) m/z (rel. int.): 192 (13.65%), 151 (11.27%), 150 (88.05%), 136 (16.40%), 135 (100.00%), 91 (15.50%), 107 (12.66%), 77 (7.18%).

4-allyl-2-methoxyphenyl butyrate (Eugenyl butyrate, EgBt). Yield, 67%: ^{1}H NMR (200 MHz, $CDCl_3$): δ (ppm) 6.97 – 6.88 (m, 1H), 6.81 – 6.71 (m, 2H), 6.08 – 5.82 (m, 1H), 5.18 – 5.03 (m, 2H), 3.81 (s, 3H), 3.38 (d, J = 6.7 Hz, 2H), 2.56 (t, J = 7.3 Hz, 2H), 1.79 (sex, J = 7.3 Hz, 2H), 1.05 (t, J = 7.4 Hz, 3H). ^{13}C NMR (50 MHz, $CDCl_3$): δ 171.9; 150.8; 138.8; 138.7; 137.0; 122.5; 120.6; 116.1; 112.6; 55.7; 40.0; 35.8; 18.5; 13.5. MS (EI) m/z (rel. int.): 234 (3.58%), 164 (100.00%), 149 (30.23%), 104 (23.40%), 103 (21.98%), 91 (40.08%), 77 (34.52%), 71 (32.80%), 43 (87.70%), 41 (40.13%).

(*E*)-2-methoxy-4-(prop-1-en-1-yl)phenyl butyrate (Isoeugenyl butyrate, IegBt). Yield, 71%: ^{1}H NMR (200 MHz, $CDCl_3$) δ (ppm) 7.05 – 6.84 (m, 3H), 6.44 – 6.30 (m, 1H), 6.18 (dq, J = 15.7; 6.3 Hz, 1H), 3.82 (s, 3H), 2.55 (t, J = 7.3 Hz, 2H), 1.88 (dd, J = 6.3; 1.2 Hz, 3H), 1.85 – 1.72 (m, 2H), 1.05 (t, J = 7.4 Hz, 3H). ^{13}C NMR (50 MHz, $CDCl_3$) δ 171.8; 151.0; 138.7; 136.9; 130.5; 125.9; 122.7; 118.4; 109.6; 55.7; 35.9; 18.5; 18.4; 13.59. MS (EI) m/z (rel. int.): 234 (2.45%), 164 (100.00%), 149 (17.41%), 103 (12.53%), 91 (23.16%), 77 (15.04%), 55 (10.84%), 43 (44.75%), 41 (19.33%).

2-isopropyl-5-methylphenyl butyrate (Thymol butyrate, TmBt). Yield, 91%: ^{1}H NMR (200 MHz, $CDCl_3$) δ (ppm) 7.20 (d, J = 7.9 Hz, 1H), 7.06 – 6.97 (m, 1H), 6.80 (d, J = 0.7 Hz, 1H), 2.96 (hept, J = 6.9 Hz, 1H), 2.56 (t, J = 7.4 Hz, 2H), 2.31 (s, 3H), 1.81 (sex, J = 7.5 Hz, 2H), 1.19 (d, J = 6.9 Hz, 6H), 1.06 (t, J = 7.4 Hz, 3H). ^{13}C NMR (50 MHz, $CDCl_3$) δ 172.4; 147.8; 137.0; 136.5; 127.0; 126.3; 122.7; 36.2; 27.0; 23.0; 20.8; 18.5; 13.7. MS

(EI) m/z (rel. int.): 220 (14.40%), 151 (10.29%), 150 (82.56%), 136 (19.09%), 135 (100.00%), 115 (7.79%), 91 (12.16%), 71 (11.12%).

5-isopropyl-2-methylphenyl butyrate (Carvacryl butyrate, CvBt). Yield, 96%: ^{1}H NMR (200 MHz, $CDCl_3$): δ (ppm) 7.14 (d, J = 7.8 Hz, 1H), 7.00 (dd, J = 7.8; 1.6 Hz, 1H), 6.85 (d, J = 1.6 Hz, 1H), 2.85 (hept, J = 6.9 Hz, 1H), 2.56 (t, J = 7.4 Hz, 2H), 2.13 (s, 3H), 1.79 (sex, J = 7.4 Hz, 2H), 1.23 (d, J = 6.9 Hz, 6H), 1.06 (t, J = 7.4 Hz, 3H). ^{13}C NMR (50 MHz, CDCl3): δ 171.9; 149.3; 148.0; 130.8; 127.1; 124.0; 119.8; 36.1; 33.5; 23.9; 18.6; 15.8; 13.7. MS (EI) m/z (rel. int.): 220 (13.63%), 151 (14.66%), 150 (97.41%), 136 (14.81%), 135 (100.00%), 91 (11.78%).

4-allyl-2-methoxyphenyl benzoate (Eugenyl benzoate, EgBz).Yield, 40%: ^{1}H NMR (200 MHz, $CDCl_3$): δ (ppm) 7.69 – 7.57 (m, 1H), 7.56 – 7.44 (m, 2H), 7.07 (dd, J = 7.4; 0.8 Hz, 1H), 6.85 – 6.78 (m, 2H), 5.99 (ddt, J = 16.9; 10.2; 6.7 Hz, 1H), 5.21 – 5.04 (m, 2H), 3.80 (s, 3H), 3.41 (d, J = 6.7 Hz, 2H). ^{13}C NMR (50 MHz, $CDCl_3$): δ 164.9; 151.0; 139.0; 138.1; 137.1; 133.4; 130.2; 129.4; 128.4; 122.6; 120.7; 116.1; 112.7; 55.8; 40.1. MS (EI) m/z (rel. int.): 268 (0.79%), 106 (7.61%), 105 (100.00%), 91 (9.96%), 78 (7.14%), 77 (78.95%), 65 (5.90%), 51 (19.08%), 41 (4.93%).

(*E*)-2-methoxy-4-(prop-1-en-1-yl)phenyl benzoate (Isoeugenyl benzoate, IegBz). Yield, 45%: ^{1}H NMR (200 MHz, $CDCl_3$) δ (ppm) 8.27 – 8.15 (m, 2H), 7.68 – 7.58 (m, 1H), 7.56 – 7.45 (m, 2H), 7.07 (d, J = 8.0 Hz, 1H), 7.00 – 6.90 (m, 2H), 6.41 (dd, J = 15.8; 1.1 Hz, 1H), 6.21 (dq, J = 15.7; 6.2 Hz, 1H), 3.82 (s, 3H), 1.89 (dd, J = 6.3; 1.2 Hz, 3H). ^{13}C NMR (50 MHz, $CDCl_3$) δ 164.8; 151.2; 138.8; 137.1; 133.4; 130.5; 130.3; 129.5; 128.5; 126.0; 122.8; 118.4; 109.8; 55.8; 18.4. MS (EI) m/z (rel. int.): 268 (13.24%), 106 (8.15%), 105 (100.0%), 91 (4.15%), 77 (28.23%).

2-isopropyl-5-methylphenyl benzoate (Thymol benzoate, TmBz). Yield, 37%: ^{1}H NMR (200 MHz, $CDCl_3$): δ (ppm) 8.22 (d, J = 7.1 Hz, 2H), 7.65 (t, J = 7.3 Hz, 1H), 7.52 (t, J = 7.3 Hz, 2H), 7.25 (d, J = 7.9 Hz, 1H), 7.06 (d, J = 7.9 Hz, 1H), 6.95 (s, 1H), 3.06 (hept, J = 6.9 Hz, 1H), 2.4 (s, 3H), 1.21 (d, J = 6.9 Hz, 6H). ^{13}C NMR (50 MHz, $CDCl_3$): δ 165.3; 148.1; 137.2; 136.6; 133.5; 130.1; 129.7; 128.6; 127.1; 126.4; 122.8; 27.3; 23.0; 20.8. MS (EI) m/z (rel. int.): 254 (2.82%), 149 (9.96%), 106 (7.62%), 105 (100.00%), 91 (5.15%), 78 (4.74%), 77 (57.07%), 51 (6.25%).

5-isopropyl-2-methylphenyl benzoate (Carvacryl benzoate, CvBz). Yield, 51%: ^{1}H NMR (200 MHz, $CDCl_3$): δ (ppm) 8.28 – 8.18 (m, 2H), 7.72 – 7.58 (m, 1H), 7.58 – 7.45 (m, 2H), 7.20 (d, J = 7.8 Hz, 1H), 7.06 (dd, J = 7.8; 1.6 Hz, 1H), 7.00 (d, J = 1.6 Hz, 1H), 2.91 (hept, J = 6.9 Hz, 1H), 2.19 (s, 3H), 1.25 (d, J = 6.9 Hz, 6H). ^{13}C NMR (50 MHz, $CDCl_3$) δ 164.9; 149.4; 148.1; 133.5; 130.9; 130.1; 129.62. 128.5; 127.3; 124.2; 119.8; 33.6; 23.9; 15.8. MS (EI) m/z (rel. int.): 254 (0.46%), 106 (6.07%), 105 (68.26%), 91 (10.43%), 79 (5.39%), 78 (8.94%), 77 (100.00%), 51 (17.75%).

The ^{1}H NMR (200MHz, $CDCl_3$) spectra for each compound are available above.

^{1}H NMR spectrum (200 MHz, CDCl3) of **4-allyl-2-methoxyphenyl acetate (Eugenyl acetate, EgAc)** with expansion of the region between 5.0 and 7.5 ppm.

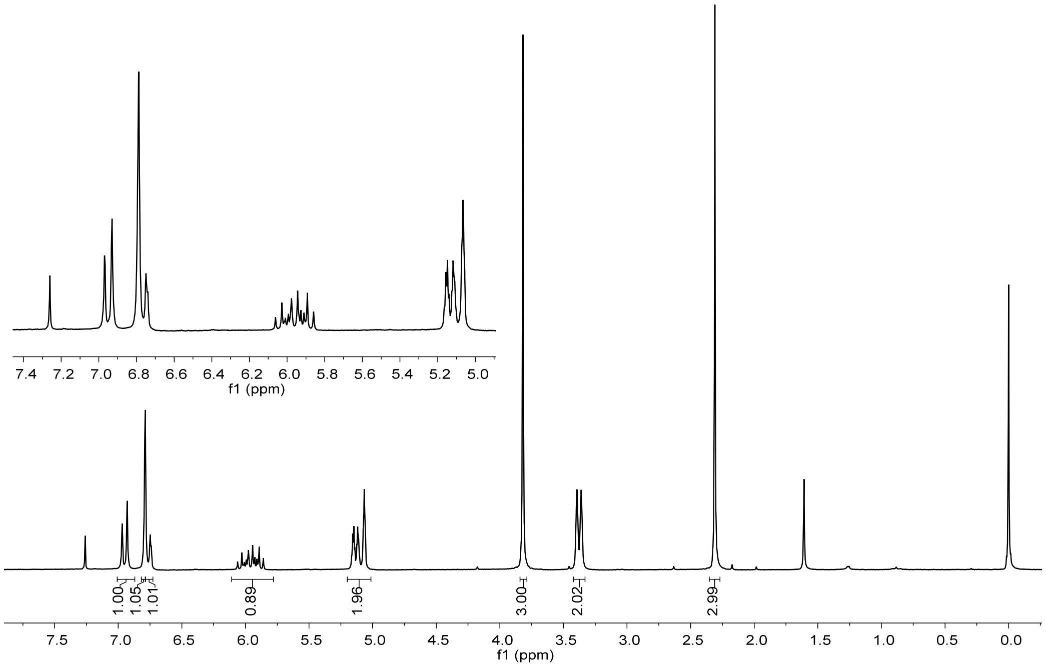

^{1}H NMR spectrum (200 MHz, CDCl3) of **4-allyl-2-methoxyphenyl butyrate (Eugenyl butyrate, EgBt)** with expansion of the region between 5.0 and 7.5 ppm.

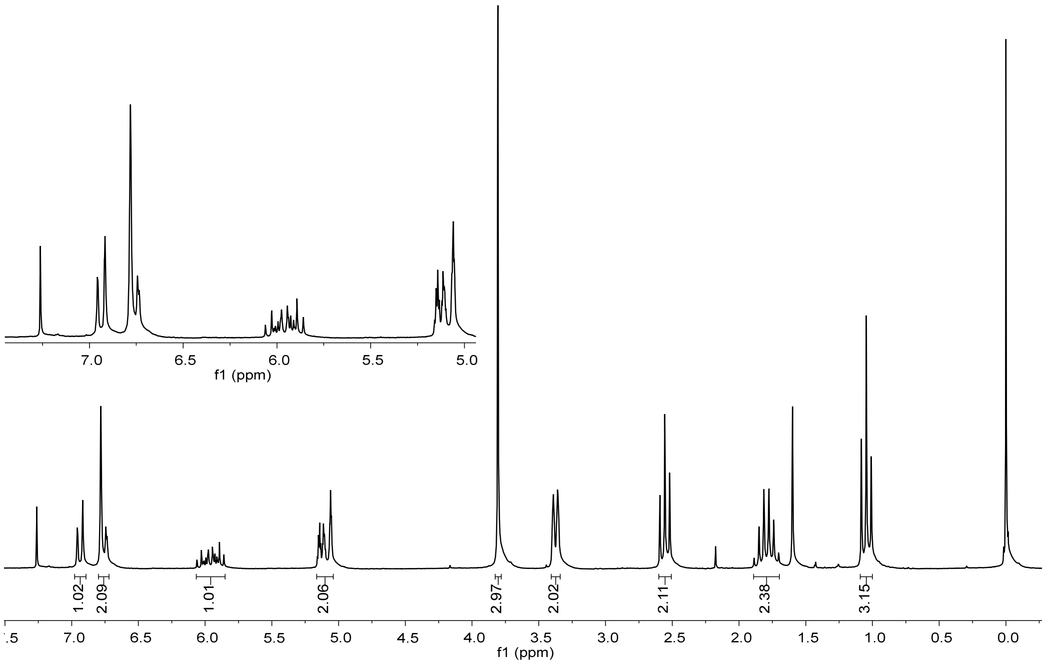

^{1}H NMR spectrum (200 MHz, CDCl3) of **4-allyl-2-methoxyphenyl benzoate (Eugenyl benzoate, EgBz)** with expansion of the region between 5.0 and 8.5 ppm.

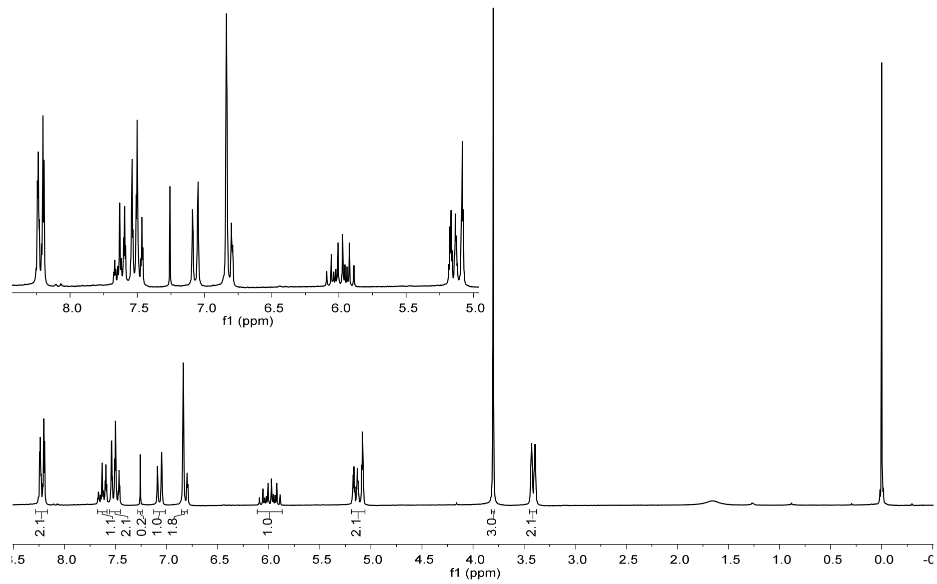

^{1}H NMR spectrum (200 MHz, CDCl3) of **(E)-2-methoxy-4-(prop-1-en-1-yl)phenyl acetate (Isoeugenyl acetate, IegAc)** with expansion of the region between 5.0 and 7.5 ppm.

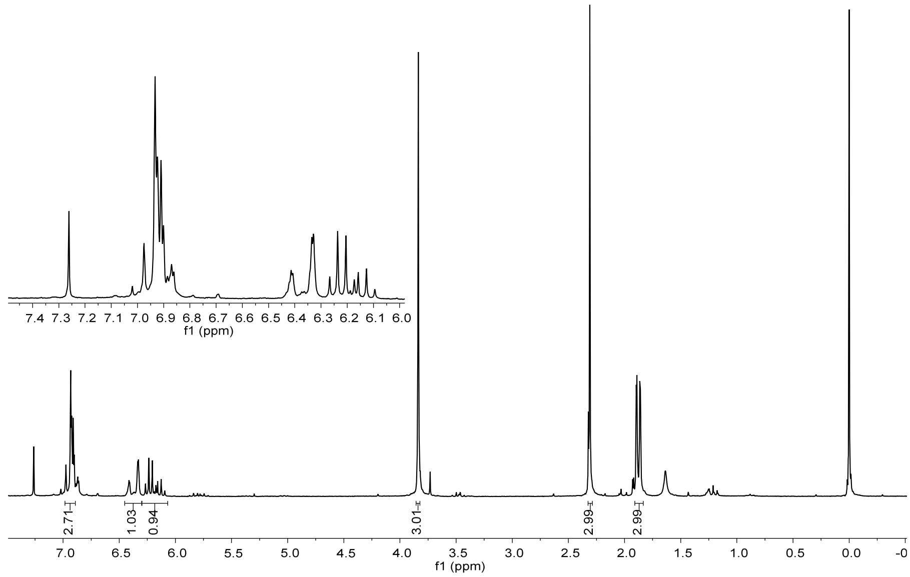

^{1}H NMR spectrum (200 MHz, CDCl3) of **(E)-2-methoxy-4-(prop-1-en-1-yl)phenyl butyrate (Isoeugenyl butyrate, IegBt)** with expansion of the region between 5.0 and 7.5 ppm.

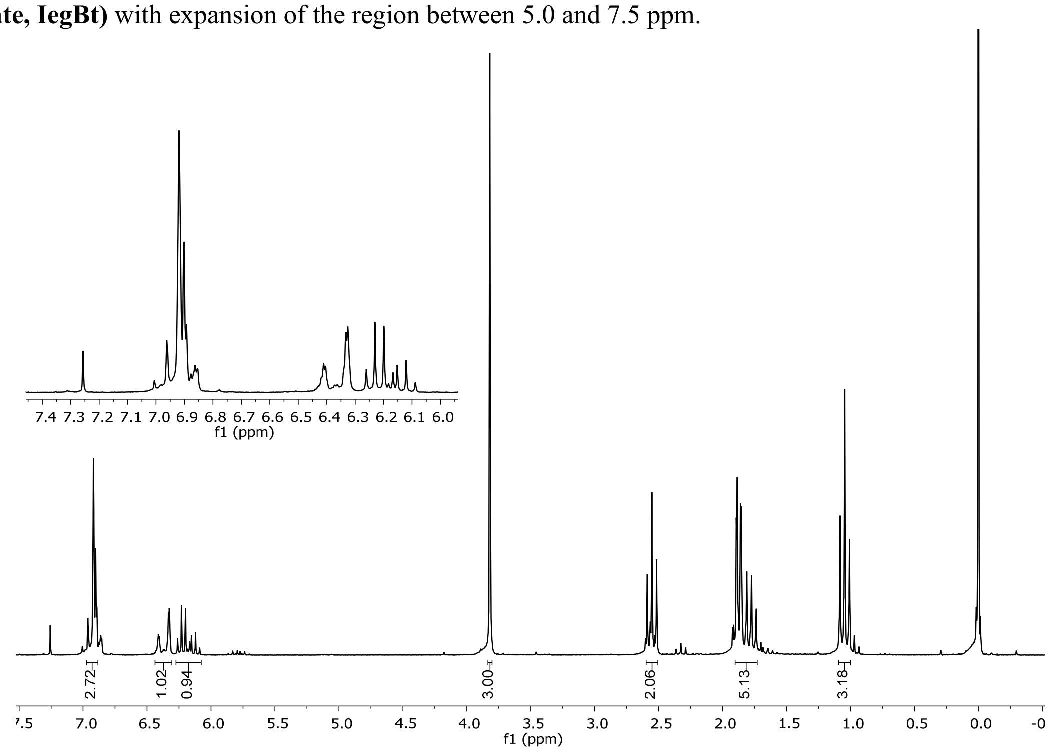

^{1}H NMR spectrum (200 MHz, CDCl3) of **(E)-2-methoxy-4-(prop-1-en-1-yl)phenyl benzoate (Isoeugenyl benzoate, IegBz)** with expansion of the region between 6.0 and 8.5 ppm.

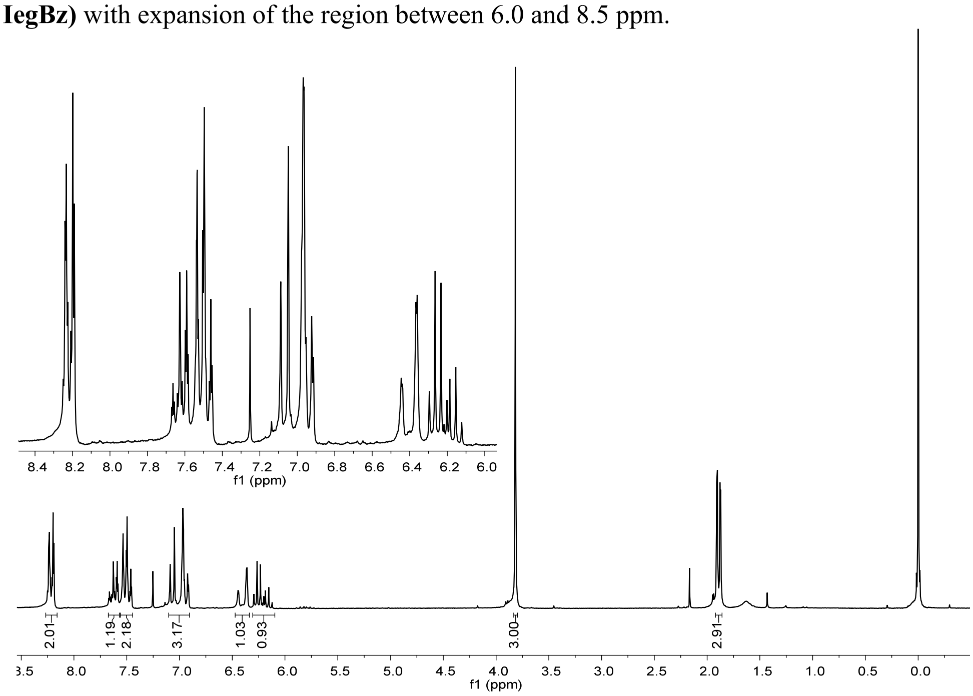

^1H NMR spectrum (200 MHz, CDCl3) of **2-isopropyl-5-methylphenyl acetate (Thymol acetate, TmAc)** with expansion of the region between 6.7 and 7.3 ppm.

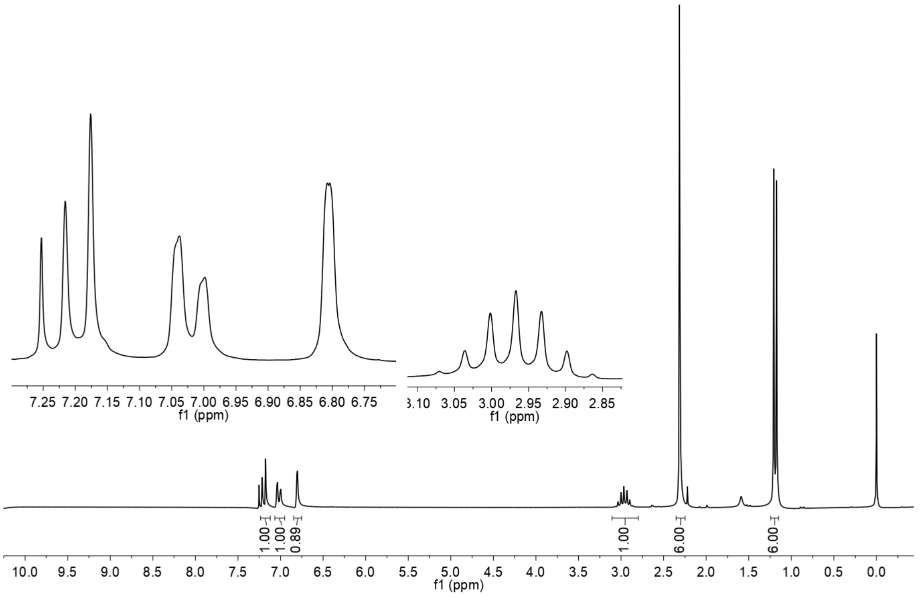

^1H NMR spectrum (200 MHz, CDCl3) of **2-isopropyl-5-methylphenyl butyrate (Thymol butyrate, TmBt)** with expansion of the region between 6.7 and 7.3 ppm.

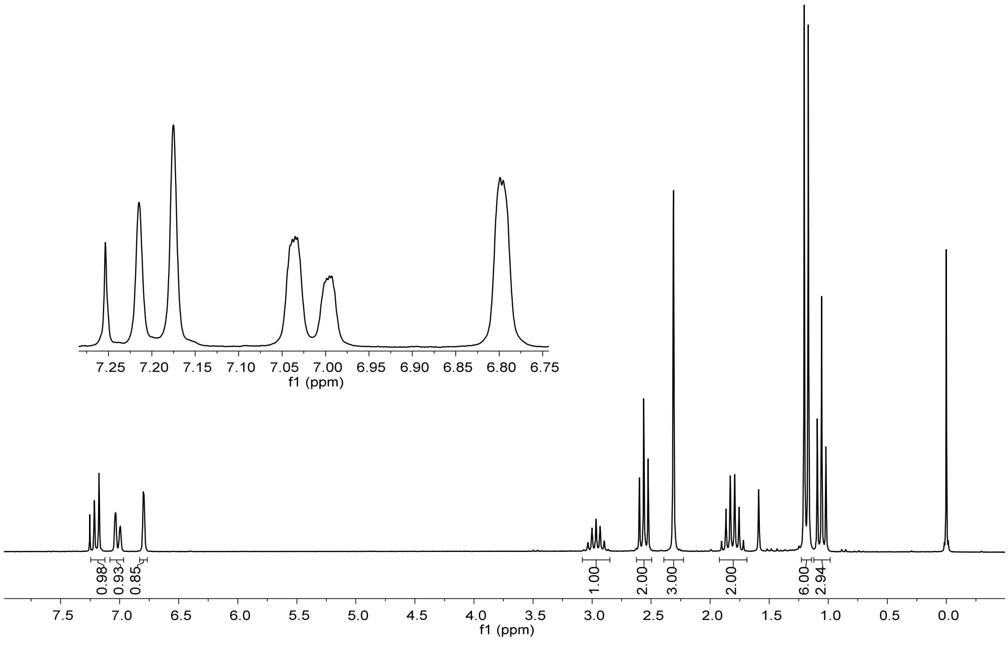

^{1}H NMR spectrum (200 MHz, CDCl3) of **2-isopropyl-5-methylphenyl benzoate (Thymol benzoate, TmBz)** with expansion of the region between 6.8 and 8.4 ppm.

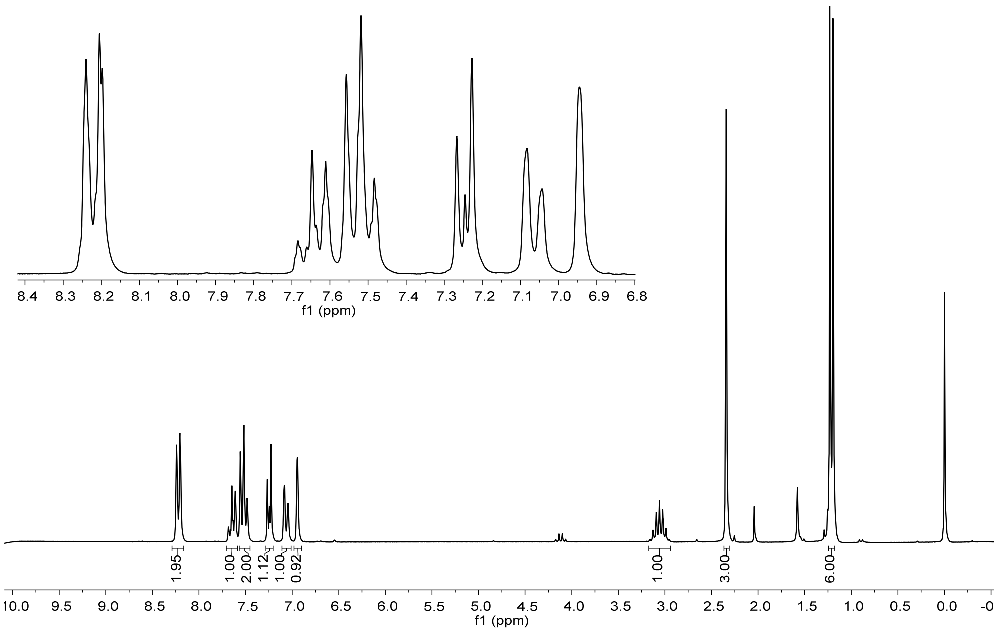

^{1}H NMR spectrum (200 MHz, CDCl3) of **5-isopropyl-2-methylphenyl acetate (Carvacryl acetate, CvAc)** with expansion of the region between 6.5 and 7.5 ppm.

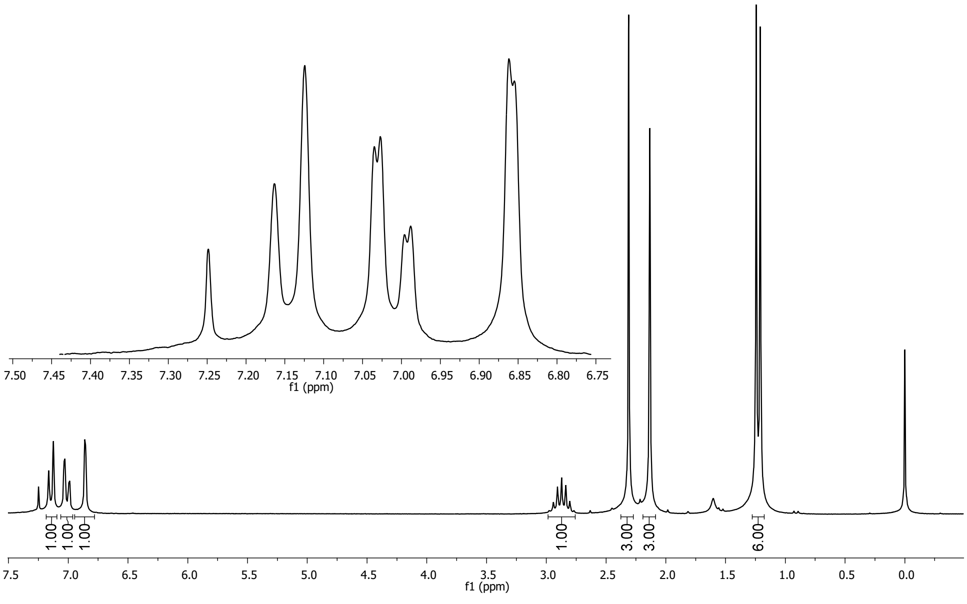

^{1}H NMR spectrum (200 MHz, CDCl3) of **5-isopropyl-2-methylphenyl butyrate (Carvacryl butyrate, CvBt)** with expansion of the region between 6.7 and 7.3 ppm.

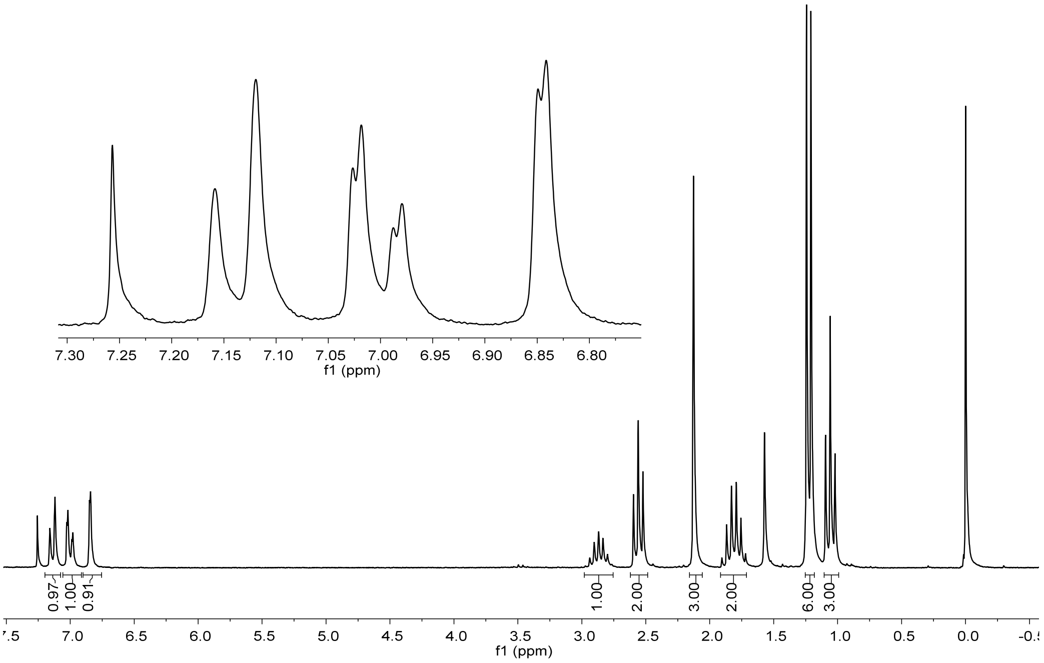

^{1}H NMR spectrum (200 MHz, CDCl3) of **5-isopropyl-2-methylphenyl benzoate (Carvacryl benzoate, CvBz)** with expansion of the region between 6.5 and 8.5 ppm.

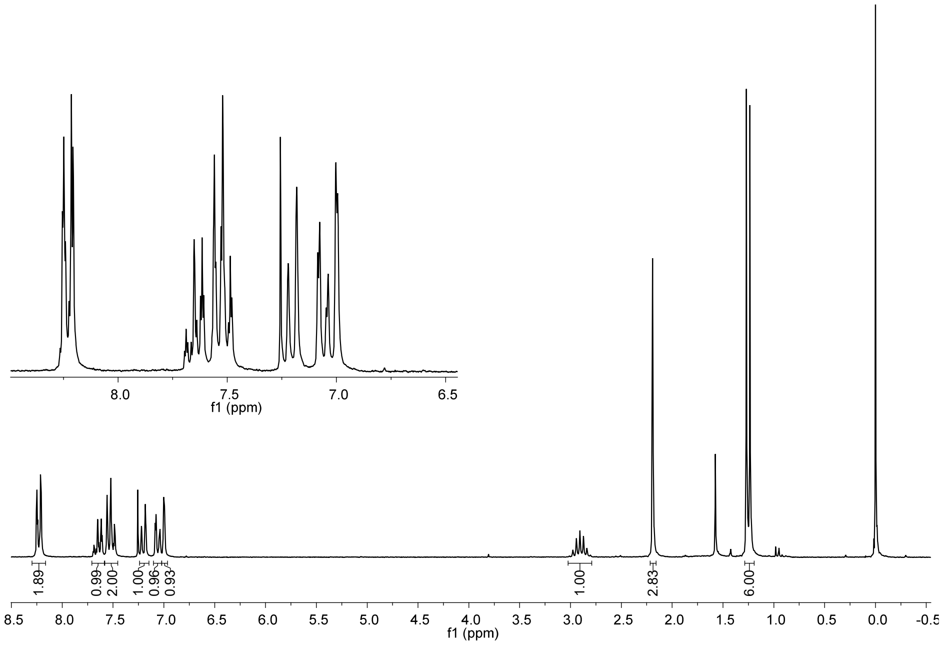

Supplement: Supplementary file 1 [file plants-13-03196-s001.zip › plants-3285510-supplementary.pdf]
